# Inhibition of Ammonia Monooxygenase from Ammonia-Oxidizing Archaea by Linear and Aromatic Alkynes

**DOI:** 10.1128/AEM.02388-19

**Published:** 2020-04-17

**Authors:** Chloë L. Wright, Arne Schatteman, Andrew T. Crombie, J. Colin Murrell, Laura E. Lehtovirta-Morley

**Affiliations:** a School of Biological Sciences, University of East Anglia, Norwich, United Kingdom; b School of Environmental Sciences, University of East Anglia, Norwich, United Kingdom; Wageningen University

**Keywords:** ammonia monooxygenase, ammonia oxidizers, inhibition, linear 1-alkynes, methanotrophs, phenylacetylene

## Abstract

Ammonia monooxygenase (AMO) is a key nitrogen-transforming enzyme belonging to the same copper-dependent membrane monooxygenase family (CuMMO) as the particulate methane monooxygenase (pMMO). The AMO from ammonia-oxidizing archaea (AOA) is very divergent from both the AMO of ammonia-oxidizing bacteria (AOB) and the pMMO from methanotrophs, and little is known about the structure or substrate range of the archaeal AMO. This study compares inhibition by C_2_ to C_8_ linear 1-alkynes of AMO from two phylogenetically distinct strains of AOA, “*Candidatus* Nitrosocosmicus franklandus” C13 and “*Candidatus* Nitrosotalea sinensis” Nd2, with AMO from Nitrosomonas europaea and pMMO from Methylococcus capsulatus (Bath). An increased sensitivity of the archaeal AMO to short-chain-length alkynes (≤C_5_) appeared to be conserved across AOA lineages. Similarities in C_2_ to C_8_ alkyne inhibition profiles between AMO from AOA and pMMO from *M. capsulatus* suggested that the archaeal AMO has a narrower substrate range than *N. europaea* AMO. Inhibition of AMO from “*Ca*. Nitrosocosmicus franklandus” and *N. europaea* by the aromatic alkyne phenylacetylene was also investigated. Kinetic data revealed that the mechanisms by which phenylacetylene inhibits “*Ca*. Nitrosocosmicus franklandus” and *N. europaea* are different, indicating differences in the AMO active site between AOA and AOB. Phenylacetylene was found to be a specific and irreversible inhibitor of AMO from “*Ca*. Nitrosocosmicus franklandus,” and it does not compete with NH_3_ for binding at the active site.

**IMPORTANCE** Archaeal and bacterial ammonia oxidizers (AOA and AOB, respectively) initiate nitrification by oxidizing ammonia to hydroxylamine, a reaction catalyzed by ammonia monooxygenase (AMO). AMO enzyme is difficult to purify in its active form, and its structure and biochemistry remain largely unexplored. The bacterial AMO and the closely related particulate methane monooxygenase (pMMO) have a broad range of hydrocarbon cooxidation substrates. This study provides insights into the AMO of previously unstudied archaeal genera, by comparing the response of the archaeal AMO, a bacterial AMO, and pMMO to inhibition by linear 1-alkynes and the aromatic alkyne, phenylacetylene. Reduced sensitivity to inhibition by larger alkynes suggests that the archaeal AMO has a narrower hydrocarbon substrate range than the bacterial AMO, as previously reported for other genera of AOA. Phenylacetylene inhibited the archaeal and bacterial AMOs at different thresholds and by different mechanisms of inhibition, highlighting structural differences between the two forms of monooxygenase.

## Abstract (precis)

Archaeal and bacterial ammonia oxidizers (AOA and AOB, respectively) initiate nitrification by oxidizing ammonia to hydroxylamine, a reaction catalyzed by ammonia monooxygenase (AMO). AMO enzyme is difficult to purify in its active form, and its structure and biochemistry remain largely unexplored. The bacterial AMO and the closely related particulate methane monooxygenase (pMMO) have a broad range of hydrocarbon cooxidation substrates. This study provides insights into the AMO of previously unstudied archaeal genera, by comparing the response of the archaeal AMO, a bacterial AMO, and pMMO to inhibition by linear 1-alkynes and the aromatic alkyne, phenylacetylene. Reduced sensitivity to inhibition by larger alkynes suggests that the archaeal AMO has a narrower hydrocarbon substrate range than the bacterial AMO, as previously reported for other genera of AOA. Phenylacetylene inhibited the archaeal and bacterial AMOs at different thresholds and by different mechanisms of inhibition, highlighting structural differences between the two forms of monooxygenase.

## INTRODUCTION

Nitrification is a key microbial process in the global nitrogen cycle. Autotrophic archaeal and bacterial ammonia oxidizers (AOA and AOB, respectively) and comammox bacteria, which carry out the complete oxidation of ammonia to nitrate (1, 2), initiate nitrification through the oxidation of ammonia (NH_3_) to hydroxylamine (NH_2_OH), a reaction catalyzed by ammonia monooxygenase (AMO). AMO is the only enzyme of the ammonia oxidation pathway which is shared by all three major groups of ammonia oxidizers (3). Quantitative assessments based on the *amoA* gene, which encodes the AmoA subunit of AMO, have revealed that AOA are ubiquitous in the environment and are among the most numerous living organisms on Earth, often outnumbering AOB in many environments where nitrification occurs (4–7). Environmental surveys using *amoA* as a marker gene have been crucial for our understanding of the distribution and diversity of AOA; however, little is known about the structure or biochemistry of the archaeal AMO and how this differs from that of AOB.

AMO is a copper-dependent multimeric transmembrane enzyme belonging to the copper-dependent membrane monooxygenase (CuMMO) superfamily, which comprises ammonia, methane, and alkane monooxygenases (7–9). Members of the CuMMO family have a broad substrate range, and it has been suggested that subsequent metabolic steps define the functional role of microbes containing CuMMO (10, 11). For example, the AOB Nitrosomonas europaea and Nitrosococcus oceani can oxidize methane but lack necessary downstream enzymes to gain reducing power from methane oxidation (12, 13). Likewise, the particulate methane monooxygenase (pMMO) of methanotrophs can cooxidize NH_3_ (14–16) as well as various hydrocarbons, for instance, linear 1-alkanes (C_2_ to C_5_) and alkenes (C_2_ to C_4_) (17–19), and halogenated hydrocarbons (20), but none of these oxidation substrates can support growth. The bacterial AMO has a broader substrate range than the pMMO and is capable of cooxidizing 1-alkanes (C_2_ to C_8_) and alkenes (C_2_ to C_5_) (21), halogenated hydrocarbons (22, 23), aromatic compounds (24), and sulfides (25, 26) to yield oxidized products. Difficulties in purifying active AMO limit the amount of structural data available, and many predictions about the structure of AMO are based on homology to the pMMO (8, 10, 27, 28). However, the pMMO itself has proven challenging to fully characterize, and the nature and location of the sites of O_2_ activation and methane oxidation remain uncertain. To date, a diiron site located on the PmoC subunit (29), and multiple copper sites of different nuclearities located on separate subunits (PmoA, PmoB, and PmoC) have all been suggested as potential active sites (27, 30–34).

Insights regarding the structure and function of AMO have largely come from whole-cell studies investigating its interaction with both reversible and irreversible inhibitors. For example, the bacterial AMO is inhibited by the copper chelator allylthiourea (ATU), which strongly indicates that it is a copper-dependent enzyme (18, 35–38). Acetylene is a well-characterized inhibitor of both AMO and pMMO (39–41). With *N. europaea*, acetylene acts as a suicide substrate, and cells require *de novo* protein synthesis of new AMO to reestablish NH_3_-oxidizing activity (42). Incubations with [^14^C]acetylene resulted in the covalent radiolabeling of *N. europaea* AMO, enabling identification of the genes coding for AMO (41, 43). A subsequent study found that the ketene product of acetylene activation bound covalently to a histidine residue (H191) in the AmoA subunit of *N. europaea*, a residue thought to be in the proximity of the AMO active site (44). While acetylene is also an irreversible inhibitor of the archaeal AMO, the AMOs from archaea lack the histidine residue responsible for binding in *N. europaea*, suggesting that the product of acetylene oxidation must bind at a different position on the enzyme. AMO from *N. europaea* is also irreversibly inhibited by other terminal and subterminal alkynes, including C_3_ to C_10_ 1-alkynes (21), 3-hexyne (45) and 1,7-octadiyne (46). Interestingly, in *N. europaea*, the degree of inhibition by 1-alkynes, as a function of chain length, inversely mirrors the activity with the corresponding 1-alkanes (21).

Virtually nothing is known about the substrate range of the archaeal AMO. Previously, Taylor et al. (47, 48) showed that in whole-cell studies, aliphatic *n*-alkynes (C_2_ to C_9_) differentially inhibited bacterial and archaeal AMOs, with AOA being less sensitive to ≥C_5_ 1-alkynes. Inhibition of AMO by 1-octyne (C_8_) has since been used in environmental and mesocosm studies to discriminate between the contributions of AOA and AOB to soil nitrification (49–52). A field study by Im et al. (53) showed that the abundance of archaeal *amoA* genes decreased when the soil was treated with the aromatic alkyne phenylacetylene, although the effects of phenylacetylene on pure cultures of AOA were not investigated. Phenylacetylene was shown to be a strong inhibitor of the AMO from *N. europaea* (41), with complete inhibition at <1 μM (54), and the AMO from *N. europaea* is capable of oxidizing aromatic compounds, including the alkane analogue of phenylacetylene, ethylbenzene (24, 55). Interestingly, the oxidation of aromatic hydrocarbons has not been observed for the pMMO (17, 21, 40, 56).

The initial aim of this study was to undertake a comprehensive assessment of the inhibition of archaeal AMO activity by C_2_ to C_8_ linear 1-alkynes using two terrestrial AOA strains from distinct thaumarchaeal lineages, “*Candidatus* Nitrosocosmicus franklandus” C13 and “*Candidatus* Nitrosotalea sinensis” Nd2. 1-Alkyne inhibition profiles of *N. europaea* AMO and the pMMO from Methylococcus capsulatus (Bath) were also investigated for comparison. For consistency and to provide a direct comparison with AMO, the inhibition of NH_3_-oxidizing activity by the pMMO from *M. capsulatus* (Bath) was investigated. NH_3_ is a cometabolic substrate of the pMMO from *M. capsulatus* (Bath) and is oxidized to hydroxylamine, which is further oxidized to produce NO_2_^−^ (14, 57).

Next, phenylacetylene inhibition profiles of NH_3_ oxidation by “*Ca*. Nitrosocosmicus franklandus” and *N. europaea* cells were compared. The kinetic mechanism of inhibition of intact cells of “*Ca*. Nitrosocosmicus franklandus” and *N. europaea* by phenylacetylene was investigated to explore differences in the biochemistry of the archaeal and bacterial AMOs. Evidence from previous studies suggests that NH_3_, rather than ammonium (NH_4_^+^), is the growth substrate oxidized by the bacterial AMO (58), but the preferred substrate (NH_3_/NH_4_^+^) oxidized by the archaeal AMO has not been determined. However, it is highly likely to also be NH_3_ based on archaeal and bacterial AMO sequence comparisons (59). At the pH of the systems used here, the majority of the NH_3_ (pK_a_ of 9.25) would be protonated. Therefore, calculations of kinetic parameters presented in this study are based on total reduced inorganic nitrogen (NH_3_ plus NH_4_^+^) as the substrate.

## RESULTS

### Sensitivity of “*Ca*. Nitrosocosmicus franklandus,” “*Ca*. Nitrosotalea sinensis,” *N. europaea*, and pMMO-expressing *M. capsulatus* (Bath) to C_2_ to C_8_ 1-alkynes.

**FIG 1:**
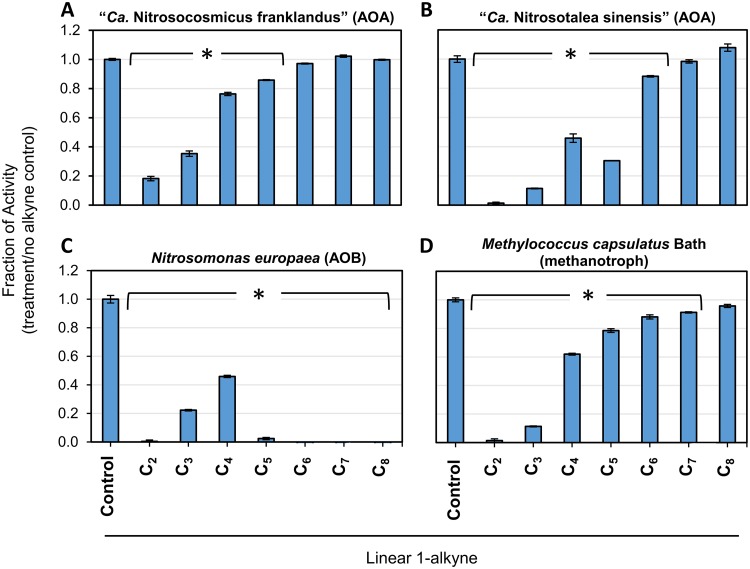
Inhibition of NO_2_^−^ production by “*Ca*. Nitrosocosmicus franklandus” (A), “*Ca*. Nitrosotalea sinensis” (B), *N. europaea* (C), and *M. capsulatus* (Bath) (D) in response to 10 μM (*C*_aq_) C_2_ to C_8_ 1-alkynes. *N. europaea*, “*Ca*. Nitrosocosmicus franklandus,” and “*Ca*. Nitrosotalea sinensis” were incubated with 1 mM NH_4_^+^ and *M. capsulatus* (Bath) with 20 mM NH_4_^+^. Error bars represent standard errors (SEs) of the means (*n* = 3). *, 1-alkyne treatments that significantly inhibited NO_2_^−^ production relative to the control treatment (*P* < 0.01).

The sensitivity of intact “*Ca*. Nitrosocosmicus franklandus” and “*Ca*. Nitrosotalea sinensis” cells to 10 μM aqueous concentrations (*C*_aq_) of C_2_ to C_8_ 1-alkynes was compared to those of *N. europaea* and the pMMO-expressing methanotroph, *M. capsulatus* (Fig. 1). NH_3_-dependent NO_2_^−^ production by both “*Ca*. Nitrosocosmicus franklandus” and “*Ca*. Nitrosotalea sinensis” was inhibited by C_2_ to C_5_ 1-alkynes (*P* < 0.001) but not by C_7_ and C_8_ (Fig. 1A and B). “*Ca*. Nitrosotalea sinensis” was strongly inhibited by C_4_ and C_5_ alkynes (degrees of inhibition, 54% ± 5% and 70% ± 1%, respectively, compared with that of controls); however, these alkynes effected only partial inhibition of NH_3_ oxidation by “*Ca*. Nitrosocosmicus franklandus” (24% ± 2% and 14% ± 1%, respectively), indicating differences in the alkyne sensitivities of different AOA strains. Additionally, 1-hexyne had a significant inhibitory effect on “*Ca*. Nitrosotalea sinensis” (*P* = 0.004) but not on “*Ca*. Nitrosocosmicus franklandus” (*P* = 0.47). NO_2_^−^ production by *N. europaea* was strongly inhibited by all 1-alkynes tested (C_2_ to C_8_). 1-Pentyne resulted in 98% ± 1% inhibition, and AMO activity was completely inhibited by C_6_ to C_8_ 1-alkynes (Fig. 1C). In the presence of C_3_ and C_4_ 1-alkynes, inhibition decreased to 78% ± 1% and 54% ± 1%, respectively. pMMO-expressing *M. capsulatus* cells oxidized NH_4_^+^ to NO_2_^−^, and NO_2_^−^ production was significantly inhibited by C_2_ to C_7_ 1-alkynes (*P* ≤ 0.001), but C_6_ and C_7_ 1-alkynes resulted in only approximately 10% inhibition compared with that of the control (Fig. 1D). NO_2_^−^ production from NH_3_ by the pMMO from *M. capsulatus* is shown in Fig. S1 in the supplemental material. The rate of NO_2_^−^ production decreased after 1 h of incubation, likely due to the toxic buildup of NO_2_^−^ and hydroxylamine in the culture.

Notably, “*Ca*. Nitrosotalea sinensis,” *N. europaea*, and *M. capsulatus* (Bath) were very sensitive to 10 μM acetylene (C_2_), with NO_2_^−^ production inhibited by >95%; however, “*Ca*. Nitrosocosmicus franklandus” appeared less sensitive to acetylene (degree of inhibition, 82% ± 3%).

### Inhibition of NO_2_^−^ production by “*Ca*. Nitrosocosmicus franklandus” and *N. europaea* in response to phenylacetylene.

**FIG 2:**
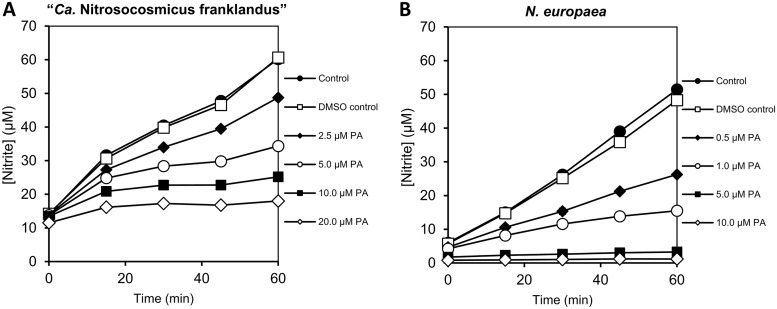
NO_2_^−^ production by “*Ca*. Nitrosocosmicus franklandus” (A) and *N. europaea* (B) in response to different concentrations of phenylacetylene (PA) dissolved in DMSO. Error bars representing SEs are included but are smaller than the markers (*n* = 3).

Given the contrasting responses of ammonia-oxidizing archaea and bacteria to linear alkynes, AMO activity in the presence of the aromatic alkyne phenylacetylene was examined in “*Ca*. Nitrosocosmicus franklandus” and *N. europaea* cells (Fig. 2). After 1 h of incubation, the rate of NH_3_-dependent NO_2_^−^ production by “*Ca*. Nitrosocosmicus franklandus” was inhibited 55.4% ± 1.4% in the presence of 5 μM phenylacetylene compared to that in the dimethyl sulfoxide (DMSO) control. Incubations in the presence of 10 and 20 μM phenylacetylene increased the inhibition to 74.7% ± 0.5% and 86.0% ± 0.4%, respectively (Fig. 2A). NO_2_^−^ production by *N. europaea* was inhibited 52.5% ± 1.7% in the presence of 0.5 μM phenylacetylene, and unlike the results from Lontoh et al. (54), who showed full inhibition at 0.6 μM, there was still partial NH_3_-oxidizing activity in the presence of 1 μM phenylacetylene (75.1% ± 1.6% inhibition on the rate of NO_2_^−^ production) (Fig. 2B). Together, the results show that “*Ca*. Nitrosocosmicus franklandus” is approximately 10× more resistant to phenylacetylene inhibition than *N. europaea*. Both “*Ca*. Nitrosocosmicus franklandus” and *N. europaea* cells incubated with 0.1% DMSO produced NO_2_^−^ at a similar rate to that of untreated controls.

### Kinetic analysis of phenylacetylene inhibition of NH_4_^+^-dependent NO_2_^−^ production by “*Ca*. Nitrosocosmicus franklandus” and *N. europaea*.

**FIG 3:**
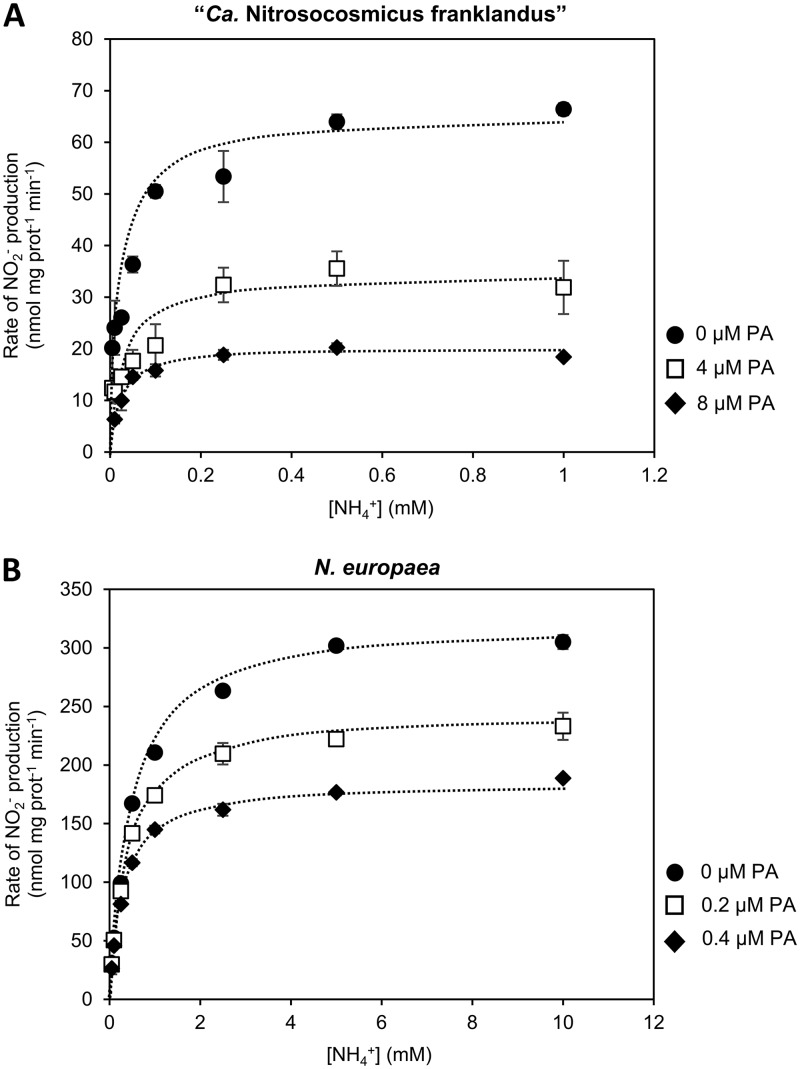
Michaelis-Menten hyperbolic plot showing the initial rate of NO_2_^−^ production by “*Ca*. Nitrosocosmicus franklandus” (A) and *N. europaea* (B) to phenylacetylene (PA) dissolved in DMSO as a function of NH_4_^+^ concentration. The *x* axis is the substrate (NH_4_^+^) concentration and the *y* axis is the initial rate of NO_2_^−^ production. Inhibition was not overcome by increasing concentration of NH_4_^+^, indicating that phenylacetylene and NH_3_ do not compete for the same binding site. Error bars represent SEs (*n* = 3).

To investigate the mode of inhibition of phenylacetylene on AMO, the initial reaction velocities of NO_2_^−^ production by “*Ca*. Nitrosocosmicus franklandus” and *N. europaea* were determined over a range of substrate (total NH_4_^+^) concentrations. The concentrations of phenylacetylene used in the kinetic analysis were selected to achieve partial inhibition of NO_2_^−^ production (Fig. 2). NH_3_-dependent kinetics of initial NO_2_^−^ production followed Michaelis-Menten-type saturation kinetics for both “*Ca*. Nitrosocosmicus franklandus” and *N. europaea* (Fig. 3A and B), where the velocity (*v*) of the AMO-catalyzed reactions was hyperbolically related to the total NH_4_^+^ concentration ([*S*]):

$$v=\frac{V_{\text{max}}.\left[S\right]}{\left(K_{m}+\left[S\right]\right)}$$

Apparent half-saturation constants for total NH_4_^+^ [*K_m_*_(app)_] and maximum velocities [*V*_max(app)_] in the presence/absence of phenylacetylene were calculated using hyperbolic regression analysis. The hyperbolic plots show that increasing the NH_4_^+^ concentration did not alleviate the inhibitory effect of phenylacetylene on NO_2_^−^ production in “*Ca*. Nitrosocosmicus franklandus” or *N. europaea* (Fig. 3A and B). This suggests that phenylacetylene is not a simple competitive inhibitor of either the archaeal or the bacterial AMO with respect to NH_3_ oxidation. Interestingly, “*Ca*. Nitrosocosmicus franklandus” and *N. europaea* seem to have different mechanisms of inhibition by phenylacetylene. With “*Ca*. Nitrosocosmicus franklandus,” the presence of 4 and 8 μM phenylacetylene decreased the *V*_max(app)_ of NO_2_^−^ production from 64.1 ± 2.6 nmol mg protein^−1 ^min^−1^ to 33.8 ± 2.2 and 20.1 ± 0.5 nmol mg protein^−1 ^min^−1^, respectively (Table 1). There was no significant change in the *K_m_*_(app)_ for cells inhibited by phenylacetylene compared to that for the control (*P* = 0.503 and *P* = 0.526 for 4 and 8 μM phenylacetylene, respectively), indicating that phenylacetylene and NH_3_ do not compete for the same binding site. Inhibition of *N. europaea* by 0.2 and 0.4 μM phenylacetylene reduced both the *K_m_*_(app)_ and the *V*_max(app)_, by approximately 30% and 40%, respectively (Table 1). This is indicative of uncompetitive inhibition and suggests that phenylacetylene binds to AMO subsequent to NH_3_ binding and at a different binding site.

**TABLE 1:** Kinetics of NH_3_-dependent NO_2_^−^ production by “*Ca*. Nitrosocosmicus franklandus” and *N. europaea* in the presence of phenylacetylene^*a*^

Strain	Phenylacetylene (μM)	*K_m_*_(app)_ (μM)	*V*_max(app)_ (nmol mg protein^−1^ min^−1^)
“*Ca*. Nitrosocosmicus franklandus”	0	26.7 (4.7)	64.1 (2.6)
	4	30.3 (8.3)	33.8 (2.2)
	8	22.9 (3.2)	20.1 (0.5)
*N. europaea*	0	520.3 (19.6)	324.4 (3.7)
	0.2	375.3 (17.4)	240.7 (2.7)
	0.4	318.4 (13.8)	188.7 (2.0)

a SEs of three replicates are in parentheses (*n* = 3).

Previously, acetylene was shown to be a competitive inhibitor of the archaeal AMO from Nitrososphaera viennensis (48). To examine if acetylene interacts competitively with “*Ca*. Nitrosocosmicus franklandus” AMO, the kinetic response of NH_3_-dependent NO_2_^−^ production by “*Ca*. Nitrosocosmicus franklandus” to 3 μM acetylene was tested using the same experimental design used to investigate phenylacetylene inhibition. In contrast to phenylacetylene, increasing the total NH_4_^+^ availability reduced acetylene inhibition, demonstrating that acetylene and NH_3_ compete for the same AMO binding site (see Fig. S2). Additionally, the *K_m_*_(app)_ increased dramatically from 18.5 ± 2.9 μM to 691.3 ± 158.1 μM NH_4_^+^ in the presence of 3 μM acetylene, but there was no change in the *V*_max(app)_ (see Table S2), also demonstrating that acetylene interacts with the NH_3_-binding site and decreases the affinity of AMO for NH_3_.

Phenylacetylene was dissolved in 100% DMSO, and all cell suspensions used in both the phenylacetylene and acetylene experiments contained 0.1% (vol/vol) DMSO. Therefore, the effect of the addition of 0.1% (vol/vol) DMSO on NH_3_ oxidation kinetics was tested separately. DMSO had no effect on kinetic parameters for NH_3_ oxidation by “*Ca*. Nitrosocosmicus franklandus.” For *N. europaea*, the presence of 0.1% (vol/vol) DMSO reduced the *K_m_*_(app)_ and *V*_max(app)_ by approximately 10% (see Table S1).

### Effect of phenylacetylene on hydroxylamine oxidation by “*Ca*. Nitrosocosmicus franklandus.”

**FIG 4:**
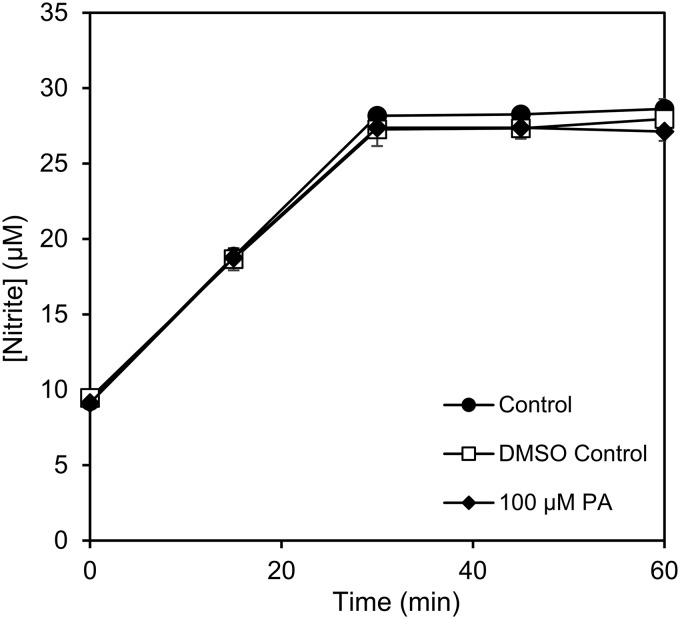
NO_2_^−^ production from hydroxylamine oxidation by “*Ca*. Nitrosocosmicus franklandus” in the presence or absence of 100 μM phenylacetylene (PA) dissolved in DMSO. Error bars represent SEs (*n* = 3).

Hydroxylamine is the product of NH_3_ oxidation by both the archaeal and bacterial AMOs and is subsequently oxidized to other intermediates in the NO_2_^−^ production pathway (60, 61). To verify that the reduction in the rate of NO_2_^−^ production by “*Ca*. Nitrosocosmicus franklandus” was due to inhibition of NH_3_ oxidation rather than the effects of downstream enzymatic reactions, we investigated hydroxylamine oxidation by “*Ca*. Nitrosocosmicus franklandus” in the presence of phenylacetylene. NO_2_^−^ production by “*Ca*. Nitrosocosmicus franklandus” was unaffected by 100 μM phenylacetylene relative to the DMSO control treatment, demonstrating that phenylacetylene is likely a specific inhibitor of the AMO from “*Ca*. Nitrosocosmicus franklandus” (Fig. 4). Hydroxylamine-dependent NO_2_^−^ production proceeded rapidly but ceased after 30 min when approximately 27 μM NO_2_^−^ had accumulated. A similar response was previously observed for the marine AOA Nitrosopumilus maritimus SCM1 (60).

### Recovery of AMO activity in “*Ca*. Nitrosocosmicus franklandus” following phenylacetylene inhibition.

**FIG 5:**
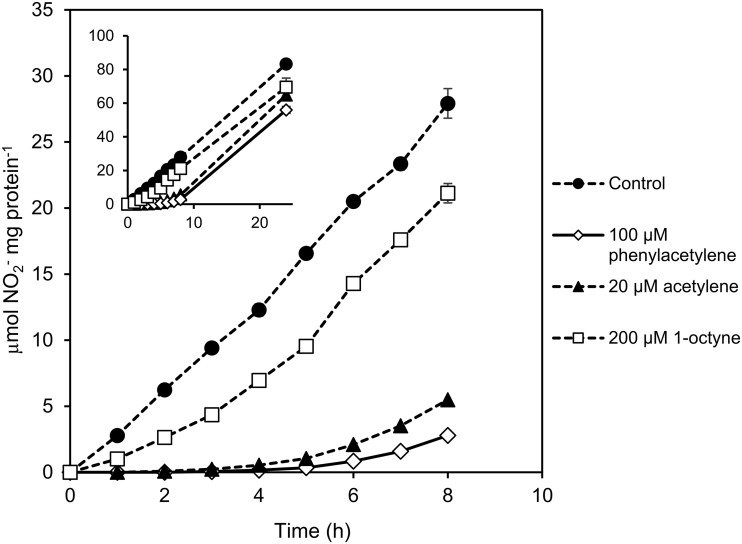
Time course of the recovery of NO_2_^−^ production by “*Ca*. Nitrosocosmicus franklandus” following overnight inhibition of NH_3_ oxidation by phenylacetylene (100 μM), acetylene (20 μM), and 1-octyne (200 μM). Error bars represent SEs (*n* = 3).

To establish whether phenylacetylene is a reversible or irreversible inhibitor of AMO from “*Ca*. Nitrosocosmicus franklandus,” the recovery of NH_3_-oxidizing activity after exposure to phenylacetylene was investigated. Previous work has shown that in order to restore AMO activity following inhibition by an irreversible inhibitor, for example, acetylene, cells need to synthesize new AMO enzyme, which results in a lag phase before activity resumes (42). “*Ca*. Nitrosocosmicus franklandus” cells were inhibited overnight by 100 μM phenylacetylene in the presence of 1 mM NH_4_^+^. Since it was previously shown that inhibition by 1-octyne was reversible in the AOA *N. viennensis*, in contrast to the irreversible action of acetylene (48), treatments with both 1-octyne and acetylene were included as controls. To ensure that the inability of cells to respond to substrate addition (NH_4_^+^) was not due to the effects of starvation, controls incubated for a similar amount of time without either inhibitor or NH_4_^+^ were included (starved cells). After the removal of the inhibitors by washing, cells were resuspended in NH_4_^+^-replete medium. NO_2_^−^ production, the proxy for NH_3_ oxidation, by “*Ca*. Nitrosocosmicus franklandus” recovered immediately following removal of 1-octyne. Cells inhibited by either acetylene or phenylacetylene had a 3- to 5-h lag time before NO_2_^−^ production began, suggesting that cells required *de novo* synthesis of new AMO in order to oxidize NH_3_ (Fig. 5). The starved cells recovered at the same rate as the controls (data not shown).

Cycloheximide is a potent inhibitor of protein synthesis in eukaryotes (62) and might be expected to have a similar effect in archaea. Previously, Vajrala et al. (63) demonstrated that it inhibited protein synthesis in the marine AOA, *N. maritimus* SCM1, preventing the recovery of NH_3_-oxidizing activity following inactivation of the AMO by acetylene. However, the same concentration range of cycloheximide did not prevent the recovery of NH_3_-oxidizing activity in *N. viennensis* following AMO inactivation with acetylene (48). Here, we observed that after complete inhibition by 20 μM acetylene, cycloheximide slowed, although it did not completely prevent, recovery of NH_3_-oxidizing activity by “*Ca*. Nitrosocosmicus franklandus” (see Fig. S3).

## DISCUSSION

### Inhibition of AMO and pMMO by linear alkynes.

Linear terminal alkynes were previously shown to differentially inhibit archaeal and bacterial AMO activity (47, 48). In agreement with this, NH_3_-dependent NO_2_^−^ production by the AOA strains “*Ca*. Nitrosocosmicus franklandus” and “*Ca*. Nitrosotalea sinensis” was considerably less sensitive to inhibition by longer-chain-length 1-alkynes (≥C_6_) compared to *N. europaea* (Fig. 1). The linear 1-alkyne inhibition profile appears to be conserved across AOA lineages, with the overall trend of increased sensitivity to short-chain alkynes and reduced sensitivity to longer-chain-length alkynes. This could indicate that, unlike the AMO from *N. europaea*, the binding cavity of the archaeal AMO cannot orientate and activate larger linear hydrocarbons such as 1-octyne, potentially due to steric hindrance caused by the bulkiness of these substrates or inhibitors. Interestingly, inhibition of the AMO from “*Ca*. Nitrosocosmicus franklandus” by 1-octyne, when used at 200 μM, was reversible, and recovery of NH_3_-oxidizing activity began immediately after removal of the inhibitor (Fig. 5). Similarly, Taylor et al. (48) showed the inhibition of AMO from *N. viennensis* by 1-octyne was also reversible.

In contrast with AOA, NH_3_ oxidation by *N. europaea* was fully or partially inhibited by all C_2_ to C_8_ 1-alkynes, with full inhibition occurring in the presence of longer-chain-length alkynes (≥C_6_). This is consistent with previous results published by Hyman et al. (21) and Taylor et al. (47) who found that long-chain-length 1-alkynes inhibited AMO of *N. europaea* more effectively than short-chain 1-alkynes. Additionally, it was observed by Hyman et al. (21) that the effectiveness of *n*-alkynes as inhibitors of AMO from *N. europaea* as the chain length increases. For example, 1-octyne inactivates *N. europaea* AMO more rapidly and effectively than shorter-chain-length 1-alkynes; however, the corresponding alkane, 1-octane, is oxidized more slowly and yields less product than short-chain alkanes (21).

The pMMO has a narrower hydrocarbon substrate range than the AMO of *N. europaea* but is capable of oxidizing short-chain *n*-alkanes (≤C_5_) and alkenes (≤C_3_) to their respective alcohols and epoxides (17). The specific site where hydrocarbon oxidation takes place within the pMMO is unclear. Intriguingly, a hydrophobic cavity identified in proximity to the predicted tricopper site in the PmoA from *M. capsulatus* (Bath) was shown to be of sufficient size to accommodate hydrocarbons of up to five carbons in length (30, 64, 65). Correspondingly, here, we found that C_2_ to C_5_ alkynes inhibited the NH_3_-oxidizing activity of pMMO from *M. capsulatus* (Bath) by more than 20%, reflecting the predicted size of this pMMO binding cavity (Fig. 1D). The inhibition of the pMMO by longer-chain alkynes (C_6_ to C_8_) was not previously tested, and we found that NH_3_ oxidation by *M. capsulatus* (Bath) was marginally inhibited by C_6_ and C_7_ alkynes, indicating that the pMMO can interact with hydrocarbons with longer chain lengths than those already known to be substrates.

The effectiveness of C_2_ to C_8_ linear 1-alkynes as inhibitors of NH_3_ oxidation by the AOA strains used in this study and in previous studies (47, 48) indicates that the archaeal AMO has a narrower hydrocarbon substrate range than the AMO of *N. europaea*. Furthermore, in terms of the 1-alkyne inhibition profile, the AMOs of “*Ca*. Nitrosocosmicus franklandus” and “*Ca*. Nitrosotalea sinensis” more closely resemble the pMMO from *M. capsulatus* (Bath) than the AMO of *N. europaea*. It could, therefore, be anticipated that the archaeal AMO oxidizes a similar range of linear *n*-alkanes and alkenes to that oxidized by the pMMO (Fig. 1).

Based on the diversity of archaeal AMO sequences (7), it is very likely that variation exists between the structure and stereoselectivity of the AMO active site from different AOA strains. Previously, Taylor et al. (47, 48) observed differences in the sensitivity of *N. maritimus*, *N. viennensis*, and “*Candidatus* Nitrososphaera gargensis” to inhibition by 1-hexyne (C_6_) and 1-heptyne (C_7_). In this study, we did not observe significant inhibition of archaeal AMO activity by 1-heptyne, although the AMO from “*Ca*. Nitrosotalea sinensis” was notably more sensitive to inhibition by C_2_ to C_5_ 1-alkynes than the AMO from “*Ca*. Nitrosocosmicus franklandus.” Additionally, 1-hexyne had a significant inhibitory effect on NO_2_^−^ production by “*Ca*. Nitrosotalea sinensis” but not by “*Ca*. Nitrosocosmicus franklandus” (Fig. 1A and B).

A considerable amount of research has focused on determining the environmental drivers influencing AOA and AOB ecology and their relative contribution to nitrification. Environmental factors, including substrate availability, pH, O_2_ availability, and temperature, have been suggested to influence the ecological niche differentiation of ammonia oxidizers and to control ammonia oxidation rates in distinct ecosystems. The resistance of “*Ca*. Nitrosocosmicus franklandus” and “*Ca*. Nitrosotalea sinensis” to inhibition by 1-octyne (C_8_) further justifies the use of 1-octyne to distinguish between AOA and AOB nitrifying activity in soils and to reveal the environmental factors influencing niche differentiation (49–51). Determining the patterns in the distributions of AOA and AOB in the environment could improve land and water management to mitigate negative impacts associated with nitrification.

### Inhibition of AMO by phenylacetylene.

Evidence from field studies indicated that phenylacetylene inhibited nitrification activity by AOA (53). Here, we examined phenylacetylene inhibition in pure culture with the terrestrial AOA strain “*Ca*. Nitrosocosmicus franklandus.” Our data show that in “*Ca*. Nitrosocosmicus franklandus,” phenylacetylene is a specific inhibitor of AMO, as it had no effect on hydroxylamine-dependent NO_2_^−^ production (Fig. 4). Kinetic analysis suggested that phenylacetylene does not compete with NH_3_ for the same AMO binding site, since increasing the substrate (NH_4_^+^) concentration did not protect against inhibition (Fig. 3A). In contrast, higher concentrations of NH_4_^+^ provided a protective effect when “*Ca*. Nitrosocosmicus franklandus” was incubated with acetylene, indicating acetylene and NH_3_ compete for the same binding site (see Fig. S2 in the supplemental material). The recovery of AMO activity following complete inhibition by phenylacetylene incorporated a significant lag phase, similar to that observed for acetylene, suggesting that inhibition by these alkynes was irreversible and that cells required *de novo* protein synthesis of new AMO to reestablish NH_3_-oxidizing activity (Fig. 5). Irreversible inhibition could indicate that the binding cavity of the AMO from “*Ca*. Nitrosocosmicus franklandus” is large enough to enable the orientation and subsequent activation of phenylacetylene and that phenylacetylene and acetylene essentially both act as suicide substrates. Curiously though, our data suggest that phenylacetylene does not interact with the same binding site on AMO as NH_3_ and acetylene.

Phenylacetylene is an irreversible inhibitor of AMO from *N. europaea* (41, 46). Here, we demonstrate that phenylacetylene does not compete with NH_3_ for the same binding site (Fig. 3B). It has been proposed that the AMO from *N. europaea* may contain two distinct binding sites, one that specifically binds NH_3_ and hydrocarbons ≤C_3_ and a second that binds larger hydrocarbons, with oxidation occurring at either site (23, 45). Alternatively, different hydrocarbons might be able to access the active site of the AMO from two different directions (45). pMMO-expressing methanotrophs also exhibit complicated inhibition patterns when exposed to multiple hydrocarbon substrates. For example, dichloromethane acted as a competitive inhibitor of methane oxidation by Methylosinus trichosporium OB3b, but trichloromethane was best described as a noncompetitive inhibitor, suggesting the existence of at least two substrate binding sites (20). Although the location and nuclearity of the active site for methane oxidation are still under debate, it is generally accepted that the pMMO contains multiple metal-binding sites, or potential active sites; therefore, it is possible that different hydrocarbons are oxidized at distinct sites on the pMMO. The noncompetitive nature of phenylacetylene inhibition, with respect to NH_3_, of the AMO from “*Ca*. Nitrosocosmicus franklandus” provides early indications either that distinct binding sites may be present on the archaeal AMO or that there are two separate routes by which substrates can access the archaeal AMO active site.

Kinetic analysis of phenylacetylene inhibition of AMO of “*Ca*. Nitrosocosmicus franklandus” and *N. europaea* revealed that phenylacetylene most likely interacts with the AMOs via distinct mechanisms. Specifically, phenylacetylene inhibition of AMO from *N. europaea* had characteristics of uncompetitive inhibition, where both the *K_m_*_(app)_ and *V*_max(app)_ decreased with increasing concentrations of phenylacetylene, indicating that the inhibitor only has affinity for the enzyme-substrate complex. Potentially, the binding of NH_3_ induces a structural change in the AMO binding cavity, enabling phenylacetylene to bind at a putative secondary (non-NH_3_) site. Phenylacetylene inhibition of the AMO from “*Ca*. Nitrosocosmicus franklandus” did not show the same characteristics as in *N. europaea* (Table 1), demonstrating that the interaction between phenylacetylene and the active site differed between the distinct AMO types.

Both AMO- and pMMO-expressing microorganisms have gained interest for their potential use in bioremediation due to their capability to cooxidize persistent organic pollutants such as halogenated alkanes and alkenes and chlorinated hydrocarbons (66, 67). Unlike the bacterial AMO, the oxidation of aromatic compounds has not been observed by the pMMO (17, 21, 45, 56). Lontoh et al. (54) showed that pMMOs from *M. capsulatus* (Bath) and several other strains of methanotrophs were relatively resistant to phenylacetylene inhibition, with whole-cell pMMO activity still present at 1 mM phenylacetylene. It is possible that aromatic compounds are simply too bulky to gain access to or be orientated at the pMMO active site (64). Although *N. europaea* appears to lack the ability to completely mineralize aromatic pollutants, it may initiate the degradation of aromatic compounds and provide oxidation products that can be transformed by other microorganisms (24). There is evidence that the archaeal AMO, unlike the pMMO, is capable of transforming aromatic compounds. Recently, Men et al. (68) demonstrated that the AOA strain “*Ca*. Nitrososphaera gargensis” was capable of cometabolizing two tertiary amines, mianserin and ranitidine, with the initial oxidative reaction most likely carried out by the AMO. Given that AOA have a significantly higher substrate affinity than AOB (69), AOA might be more effective in the biotransformation of some organic pollutants.

This research offers new insights into the structures and substrate ranges of AMOs from archaea using alkyne inhibitors in comparison with that of other members of the CuMMO family. Future studies should investigate the inhibitory effect and subsequent cooxidation of potential archaeal AMO substrates. Examining alternative substrate reactions and products could provide information about archaeal AMO stereoselectivity, advance our understanding of the enzyme structure, and improve predicted structural models for archaeal AMO.

## MATERIALS AND METHODS

### Materials.

Phenylacetylene (98%) and propyne, 1-pentyne, 1-hexyne, 1-heptyne, and 1-octyne (C_3_, C_5_, C_6_, C_7_, and C_8_ linear 1-alkynes, respectively, ≥97%) were obtained from Sigma-Aldrich. 1-Butyne was supplied by Apollo Gases Ltd. Acetylene was obtained from BOC, a member of the Linde Group. Protein concentrations were determined using a Pierce bicinchoninic acid (BCA) protein assay kit (Thermo Scientific) as described by the manufacturer.

### Growth of cultures.

“*Candidatus* Nitrosotalea sinensis” Nd2 and “*Candidatus* Nitrosocosmicus franklandus” C13 (70, 71) were grown as follows. “*Ca*. Nitrosocosmicus franklandus” was cultivated in freshwater medium (FWM) buffered with 10 mM HEPES (pH 7.5) and supplemented with 4 mM NH_4_Cl as previously described (71). The acidophilic AOA “*Ca*. Nitrosotalea sinensis” was cultivated in FWM buffered with 2.5 mM morpholineethanesulfonic acid (MES; pH 5.3) and supplemented with 400 μM NH_4_Cl as previously described (70). Both “*Ca*. Nitrosocosmicus franklandus” and “*Ca*. Nitrosotalea sinensis” were grown in 800-ml volumes in 1-liter Duran bottles incubated statically in the dark at 37°C. Nitrosomonas europaea ATCC 19718 was obtained from the University of Aberdeen culture collection and cultivated in 200-ml volumes, in 500-ml conical flasks, shaking (160 rpm) at 30°C in modified Skinner and Walker (72) medium (pH ∼7.5) containing 0.235 g liter^−1^ (NH_4_)_2_SO_4_, 0.2 g liter^−1^ KH_2_PO_4_, 0.04 g liter^−1^ CaCl_2_·2H_2_O, 0.04 g liter^−1^ MgSO·7H_2_O, and 0.3 mg liter^−1^ FeNa-EDTA, buffered with 10 mM HEPES (pH 7.5), and 5% (wt/vol) Na_2_CO_3_. Methylococcus capsulatus (Bath) was grown in 50-ml volumes in 250-ml Quickfit conical flasks, shaking (180 rpm) at 37°C in nitrate mineral salts (NMS) supplemented with 20 μM copper to promote pMMO expression under a CH_4_ atmosphere of 40%. To confirm that *M. capsulatus* cells were only expressing pMMO and not soluble MMO (sMMO), the naphthalene assay, which is specific for sMMO activity, was used (73) with sMMO-expressing Methylocella silvestris cells as positive controls. The AOA strains “*Ca*. Nitrosocosmicus franklandus” and “*Ca*. Nitrosotalea sinensis” are available upon request.

### Nitrite assay.

NO_2_^−^ concentrations were determined colorimetrically in a 96-well format using Griess reagent as previously described (70). Absorbance measurements were performed at a 540-nm wavelength using a VersaMax microplate reader (Molecular Devices).

### Inhibition of whole cells by alkynes.

“*Ca*. Nitrosocosmicus franklandus” and “*Ca*. Nitrosotalea sinensis” were cultivated to mid-exponential phase (∼600 to 700 μM and ∼80 to 90 μM NO_2_^−^ accumulated, respectively), and 1,600 ml was harvested by filtration onto nucleopore 0.2-μm membrane filters (PALL). “*Ca*. Nitrosocosmicus franklandus” cells were washed and resuspended in 200 ml 10 mM HEPES (pH 7)-buffered FWM salts to ∼2 × 10^7^ cells/ml. “*Ca*. Nitrosotalea sinensis” cells were washed and resuspended in 100 ml 2.5 mM MES (pH 5.3)-buffered FWM salts to ∼3 × 10^7^ cells/ml. *N. europaea* was grown to mid-exponential phase, and a 400-ml culture was harvested by filtration, washed, and resuspended to ∼3 × 10^7^ cells/ml in 200 ml 50 mM sodium phosphate buffer (pH 7.7) containing 2 mM MgCl_2_ (12). *M. capsulatus* cells were grown to an optical density at 540 nm (OD_540_) of 0.8, and 100 ml was harvested by centrifugation (14,000 × *g*, 10 min). Cells were washed and resuspended in 50 ml 10 mM piperazine-*N*,*N*′-bis(2-ethanesulfonic acid) (PIPES) buffer (pH 7) to ∼2 × 10^8^ cells/ml. Cells were rested for 1 h at their respective growth temperatures to achieve a baseline for enzyme activity assays. Aliquots of 5 ml “*Ca*. Nitrosocosmicus franklandus,” *N. europaea*, and *M. capsulatus* and 4 ml “*Ca*. Nitrosotalea sinensis” cell suspension were added to acid-washed 23-ml glass vials, which were then sealed with gray butyl rubber stoppers which had been autoclaved two times to remove contaminating substances. C_2_ to C_8_ linear 1-alkynes were added to the headspace as vapor to achieve a 10 μM aqueous concentration (*C*_aq_), calculated using the Henry’s law coefficients obtained from Sander (74). Phenylacetylene was dissolved in 100% dimethyl sulfoxide (DMSO) to achieve various stock solutions. A final volume of 5 μl stock solution was added to cell suspensions, resulting in 0.1% (vol/vol) DMSO plus the desired concentration of phenylacetylene. Preliminary experiments determined that the addition of 0.1% (vol/vol) DMSO did not affect NH_4_^+^-oxidizing activity (data not shown), and control treatments containing 0.1% (vol/vol) DMSO without phenylacetylene or acetylene were included. Cells were preincubated with inhibitors for 30 min to allow for the gas-liquid phase partitioning of the alkynes, at 37°C for “*Ca*. Nitrosocosmicus franklandus,” “*Ca*. Nitrosotalea sinensis,” and *M. capsulatus* and at 30°C for *N. europaea*. Total inorganic ammonium (NH_3_ plus NH_4_^+^), referred to as NH_4_^+^, was then added as NH_4_Cl or (NH_4_)_2_SO_4_ (reflecting the growth medium) to initiate NH_3_-oxidizing activity, and vials were incubated at the respective growth temperatures of the microorganisms. *M. capsulatus* was incubated with shaking (150 rpm). AMO and pMMO activity was determined by assaying NO_2_^−^ production from NH_3_ oxidation. NO_2_^−^ production was measured and quantified as described above by withdrawing a sample of culture through the septum every 15 min for 2 h unless otherwise stated. All treatments were carried out in triplicates, and experiments were performed at least three times with similar results.

### Sensitivity of isolates to C_2_ to C_8_ 1-alkynes.

C_2_ to C_8_ linear 1-alkynes were added to vials using a gas tight syringe. To initiate NH_3_ oxidation by “*Ca*. Nitrosocosmicus franklandus,” *N. europaea*, and “*Ca*. Nitrosotalea sinensis,” NH_4_^+^ was added to a concentration of 1 mM by injection through the septum. For *M. capsulatus* (Bath), sodium formate was added first, as a source of reductant, immediately followed by NH_4_^+^, both at a final concentration of 20 mM.

### Sensitivity of “*Ca*. Nitrosocosmicus franklandus” and *N. europaea* to phenylacetylene.

Phenylacetylene was added to achieve concentrations ranging from 2.5 to 20 μM for “*Ca*. Nitrosocosmicus franklandus” and 0.5 to 10 μM for *N. europaea*. To initiate ammonia oxidation, NH_4_^+^ was added to final concentrations of 0.5 mM and 5 mM to “*Ca*. Nitrosocosmicus franklandus” and *N. europaea*, respectively. NO_2_^−^ production was measured for 60 min.

### Relationship between NH_4_^+^ oxidation and phenylacetylene inhibition kinetics of “*Ca*. Nitrosocosmicus franklandus” and *N. europaea*.

To determine NH_3_ oxidation kinetics in the presence of phenylacetylene, “*Ca*. Nitrosocosmicus franklandus” and *N. europaea* cells were harvested and resuspended as described above, but to final concentrations of 1 × 10^7^ and 8 × 10^6^ cells/ml, respectively. “*Ca*. Nitrosocosmicus franklandus” cell suspensions were preincubated with phenylacetylene (0, 4, or 8 μM) or acetylene (0 or 3 μM) for 30 min before the addition of various concentrations of NH_4_^+^ (0.005 to 1 mM). *N. europaea* cell suspensions were preincubated with phenylacetylene (0, 0.2, or 0.4 μM) before the addition of 0.05 to 10 mM NH_4_^+^. Additional experiments were carried out to test the effect of 0.1% (vol/vol) DMSO on NH_3_ oxidation kinetics by “*Ca*. Nitrosocosmicus franklandus” and *N. europaea* (see Table S1 in the supplemental material).

### Phenylacetylene inhibition of hydroxylamine oxidation by “*Ca*. Nitrosocosmicus franklandus.”

“*Ca*. Nitrosocosmicus franklandus” cell suspensions were incubated with 0 or 100 μM phenylacetylene. Hydroxylamine was added at a concentration of 200 μM, and hydroxylamine-dependent NO_2_^−^ production was measured over 60 min as described above.

### Recovery of AMO activity from “*Ca*. Nitrosocosmicus franklandus” following phenylacetylene inhibition.

“*Ca*. Nitrosocosmicus franklandus” cells were grown to mid-exponential phase, and 3,200 ml was harvested by filtration as described above and concentrated into 70 ml FWM containing 10 mM HEPES (pH 7.5). Aliquots of 5 ml cell suspension were added to glass vials and sealed with butyl rubber seals. Phenylacetylene (100 μM) and 1-octyne (200 μM) were added from DMSO stock solutions (as described above), and acetylene (20 μM) was added from a 1% (vol/vol in air) gaseous stock. Both control and acetylene treatments also contained 0.1% (vol/vol) DMSO. The addition of NH_4_^+^ (1 mM) initiated NH_3_-oxidizing activity and vials were incubated at 37°C overnight (16 h). NO_2_^−^ production was monitored for 1 h to assess baseline activity. To remove inhibitors and test AMO recovery, samples were pooled into 50-ml Falcon tubes, and the cells were washed three times in FWM containing 10 mM HEPES (pH 7.5) by centrifugation (12,000 × *g* for 10 min at 5°C). The pellet was resuspended in 700 μl FWM containing 10 mM HEPES (pH 7.5). Aliquots (200 μl) of cell suspension were added to 4.8 ml FWM containing 10 mM HEPES (pH 7.5) plus 1 mM NH_4_^+^, resulting in a final cell concentration of ∼1.3 × 10^7^ cells/ml. Vials were incubated in a water bath (37°C), and NO_2_^−^ production was monitored over 24 h.

### Statistics.

Linear 1-alkyne data were plotted as average activity as a fraction of the control treatments (no inhibitor). To analyze phenylacetylene inhibition kinetics, the initial rates of NO_2_^−^ production were plotted against NH_4_^+^ concentration. A nonlinear regression was used to estimate the *K_m_*_(app)_ and *V*_max(app)_ for NH_4_^+^ using the Hyper32 kinetics package. Significant differences between treatments were identified by one-way analysis of variance (ANOVA) with Dunnett’s (2-sided) *post hoc* test (IBM SPSS version 25).

## Supplementary Material

Supplemental file 1
