# Supplementary material for: Inhibition of Ammonia Monooxygenase from Ammonia-Oxidizing Archaea by Linear and Aromatic Alkynes
Source: Appl Environ Microbiol. 2020 Apr 17;86(9):e02388-19. doi: 10.1128/AEM.02388-19 (PMC7170481; doi:10.1128/AEM.02388-19)
Supplement: Supplemental file 1 [file AEM.02388-19-s0001.pdf]

Supplementary data

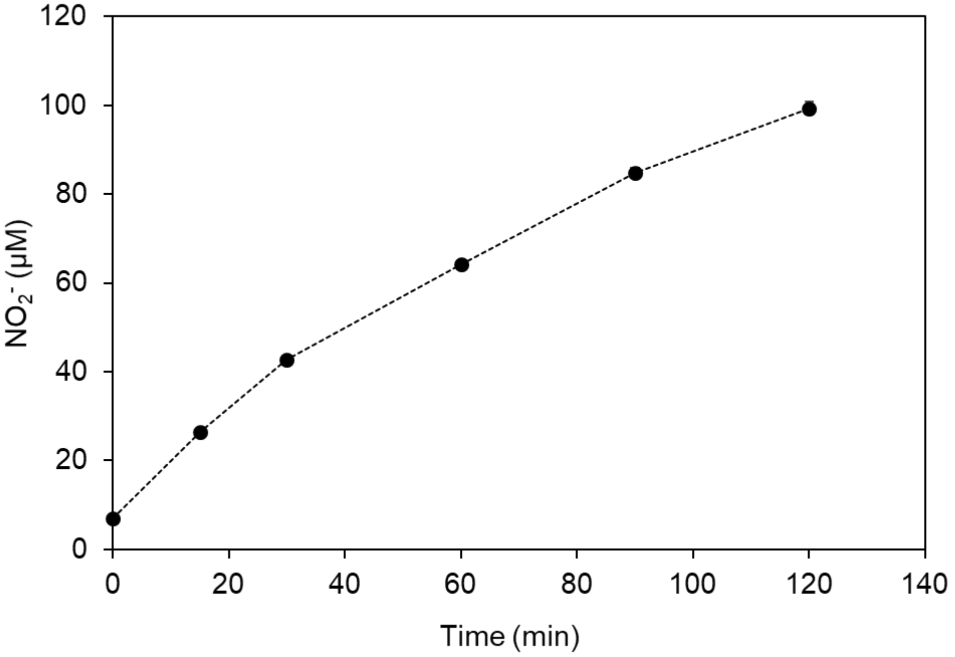

FIG S1 NO_2^- production by *M. capsulatus* (Bath) in response to 20 mM NH_4Cl and 20 mM sodium formate as a reductant. Error bars representing SE are not visible (n = 3).

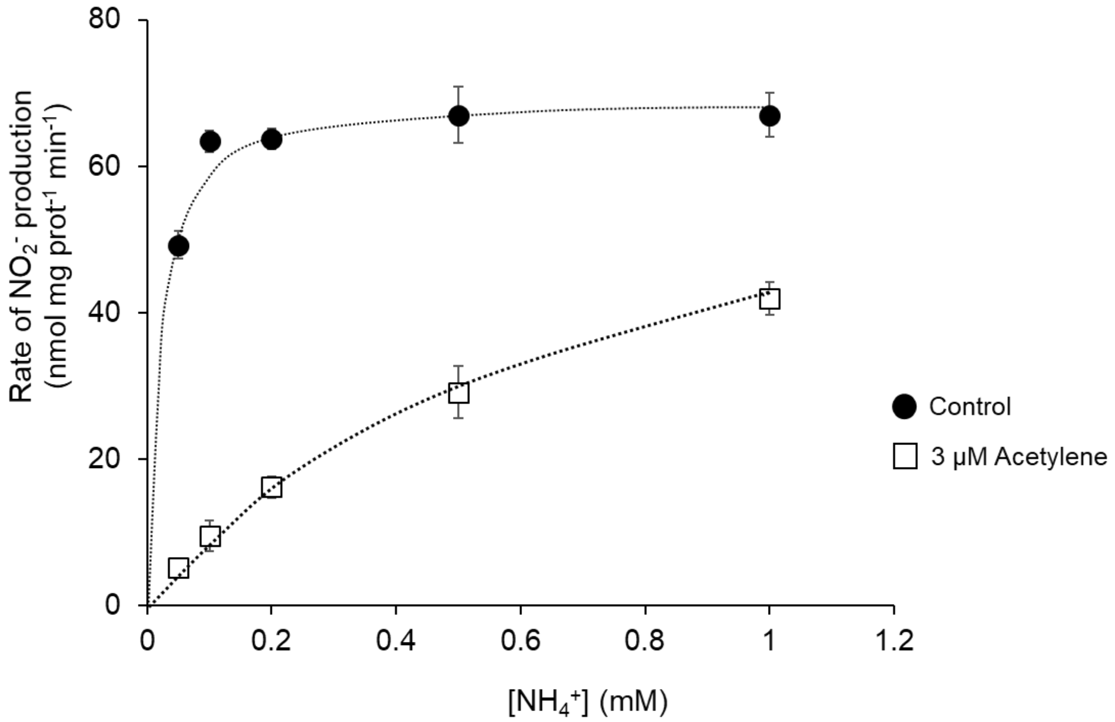

FIG S2 Michaelis-Menten hyperbolic plot showing the initial rate of NO_2^- production by "*Ca*. Nitrosocosmicus franklandus" with acetylene (3 μM) as a function of NH_4^+ concentration. Increasing the concentration of NH_4^+ reduced the effectiveness of acetylene as an inhibitor of the AMO and increased the rate of NO_2^- production, indicating acetylene competes with NH_3 for the same binding site. All replicates contained 0.1% (v/v) DMSO for direct comparison with the phenylacetylene data. Error bars represent SE (n = 3).

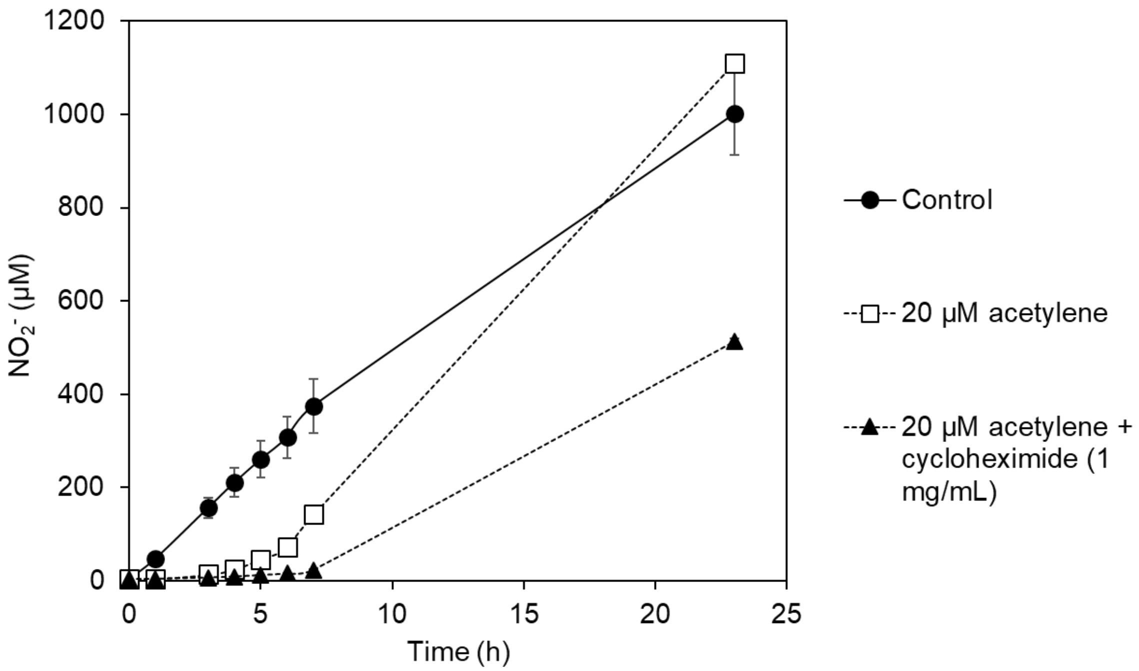

FIG S3 Time course recovery of NO_2^- production by "*Ca*. Nitrosocosmicus franklandus" following overnight inhibition of NH_3 oxidation by acetylene (20 µM). The pre-treatment to inactivate the AMO by acetylene was carried out as stated in the methods (main text). After washing and resuspending cells in fresh medium, cycloheximide at a final concentration of 1 mg/mL was added to acetylene-treated cells. Eventually, NO_2^- production recovered in cycloheximide-treated cells, reading approximately 475 µM NO_2^- compare with 1000 µM in control and acetylene-only cells at 24 h. Error bars represent SE (n = 3).

Table S1 Kinetics of NH_3-dependent NO_2^- production by "*Ca*. Nitrosocosmicus franklandus" and *N. europaea* in the presence of 0.1% DMSO. SE of three replicates are in parentheses (n=3).

Strain	Treatment	$K_{m(app)}$ (μM)	$V_{max(app)}$ (nmol NO_2^- mg protein min^{-1})
"*Ca.* Nitrosocosmicus franklandus"	Control	21.1 (3.14)	69.8 (2.6)
	0.1% DMSO	20.1 (1.8)	69.6 (1.5)
N. europaea	Control	537.6 (26.7)	375.3 (4.4)
	0.1% DMSO	474.8 (18.8)	329.2 (3.3)

Table S2 Kinetics of NH_3-dependent NO_2^- production by "*Ca*. Nitrosocosmicus franklandus" in the presence of acetylene and 0.1% (v/v) DMSO. SE of three replicates are in parentheses (n=3).

Acetylene (µM)	$K_{m(app)}$ (µM)	$V_{max(app)}$ (nmol mg prot^{-1} min^{-1})
0	18.5 (2.9)	69.6 (1.6)
3	691.3 (158.1)	69.4 (11.7)
